# OCTA biomarkers in adults aged 50 and above: a prospective and cross-sectional community-based study

**DOI:** 10.1186/s12886-023-02815-6

**Published:** 2023-02-16

**Authors:** Yan Kiu Li, Nicholas Siu-Kay Fung, Jonathan C.H Chan, Bonnie N.K. Choy, Loraine L.W. Chow, Kendrick C. Shih, Jasper K.W. Wong, Ian Y.H. Wong

**Affiliations:** 1 LKS Faculty of Medicine, University of Hong Kong, Hong Kong, China; 2 Department of Ophthalmology, Li Ka Shing Faculty of Medicine, School of Clinical Medicine, University of Hong Kong, Room 301, Level 3, Block B, Cyberport 4, 100 Cyberport Road, Pokfulam, Hong Kong China; 3 Department of Ophthalmology, Hong Kong Sanatorium and Hospital, Happy Valley, Hong Kong

**Keywords:** Epidemiology, OCTA, Macula, Vision

## Abstract

**Background/Aims:**

To assess the normative values and parameters of optical coherence tomography angiography (OCTA) influencing the best corrected visual acuity (BCVA) in adults aged 50 and above.

**Methods:**

This was a prospective cross-sectional study from an eye screening programme in Hong Kong for 4188 citizens aged 50 and above. Images were analysed using a validated quantification software calculating vessel density and capillary perfusion density (CPD), along with other OCTA parameters, such as the foveal avascular zone area (FAZ) and circularity. OCTA data was collected from May 2019 to December 2020, including a total of 4188 healthy eyes from 4188 subjects.

**Results:**

Mean superficial vessel density (MSVD) was 14.48 ± 3.60 mm^− 1^, while the mean capillary perfusion density (MCPD) was 0.41 ± 0.06. Multivariate analysis revealed ageing (β = 0.321, p < 0.001), being male (β=-0.089, p < 0.001), having a high body mass index (BMI) (β = 0.039, p = 0.006), high FAZ area and low FAZ circularity (β = 0.039 and − 0.034, p = 0.01 and 0.024 respectively), low MSVD in the outer ring (β=-0.513, p < 0.001), specifically in the nasal and temporal outer quadrants (β = -0.226 and − 0.259, p < 0.001 for both), and low MCPD in the outer superior quadrant (β= -0.123, p = 0.016) being independently associated with BCVA.

**Conclusion:**

High FAZ area and low FAZ circularity, low MSVD in the outer ring, specifically the nasal and temporal outer quadrants, and low MCPD in the outer superior quadrant can be used as biomarkers in predicting a low visual acuity in adults aged 50 and above.

## Introduction

Adequate perfusion of the retina is crucial for its homeostasis and for optimal visual function. Optical coherence tomography angiography (OCTA) has recently gained popularity in the detection of retinal microvascular abnormalities, due to its non-invasive and quantitative nature, and is the preferred modality for screening compared to fluoresceine angiography (FA), which visualizes retinal blood flow dynamics through the intravenous injection of fluorescein dye [1] and can be associated with common adverse effects such as nausea and skin irritations [2, 3]. These adverse effects are not seen with OCTA, which is non-invasive and can provide cross-sectional three-dimensional (3D) scans of the macular cube layers, enabling quantitative analysis of the superficial capillary plexus (SCP), deep capillary plexus (DCP), choroidal/optic nerve head (ONH) microvasculature, and the foveal avascular zone (FAZ), using motion contrast imaging, with FAZ circularity measured by as an index with the formula 4π (area/perimeter^2^) [4]. Previous studies have demonstrated OCTA’s high inter-visit reproducibility and intra-visit repeatability [5, 6], and its ability to remove projection artifacts through an inbuilt algorithm [7] to improve visualization and quantification of retinal vessels.

However, few studies have assessed the associations of OCTA biomarkers, especially the mean superficial vessel density (MSVD) and mean capillary perfusion density (MCPD) on visual acuity (VA), which is crucial in identifying risk factors for reduced visual function, especially among the aged population of 50 and above, who are at a higher risk of developing degenerative visual disorders. Furthermore, although studies have been conducted on the effect of age and myopia on SCP values [8, 9], most are limited by small sample sizes coupled with widely varying age groups, such as from 18–85 [9], 18–82 [10], and 30–74 [11], and none have identified the specific OCTA parameters associated with VA. A robust normative database for a large population of age 50 or above, when most retinopathies are diagnosed, also remains lacking. A larger sample of subjects recruited from this specific population aged 50 and above can mitigate the selection bias inherent in studies with small sample size and/or subjects recruited from clinical settings. In this study, we aim to determine the normative values, as well as systemic factors and OCTA biomarkers predictive of best corrected visual acuity (BCVA) in subjects age 50 or above who are recruited through a large prospective, cross-sectional and community-based study.

## Methods

### Study design and patient selection

We collected subject data from the Southern District Signature Project Scheme (SDSPS), which in collaboration with the Department of Ophthalmology at the University of Hong Kong, offers complimentary eye examination to all residents of the Southern District of Hong Kong aged 50 or above during the study period from May 2019 to December 2020 on a voluntary basis. The study was approved by the Institutional Review Board of the University of Hong Kong and Hospital Authority Hong Kong West cluster (HKU / HA HWC IRB ref. UW19-440) and conformed to the Declaration of Helsinki. All participants provided written informed consent for use of their anonymized data for this research.

The inclusion criteria encompassed: subjects aged 50 or above, with a BCVA logarithm of the minimum angle of resolution (BCVA LogMAR) ≤ 0.8 (Snellen chart 20/125), derived from previous studies revealing that this threshold included a sufficient number of people with eyes of acceptable VA. Vision was less than 20/20 even in normal patients, as most of the population in Hong Kong do suffer from myopia, and degenerative changes commonly occur with an advanced age, even in patients without macular/retinal pathologies, such as presbyopia. The exclusion criteria were: incomplete/poor quality scans, macular/retinal pathologies, such as macular edema, diabetic retinopathy (DR), retinal artery/vein occlusion, epiretinal membranes, exudative age-related macular degeneration (AMD), retinal pigment epithelium atrophy, and retinal hemorrhage, spherical equivalence over 6.0 diopters, as well as previous intraocular surgeries including pars plana vitrectomy (PPV), globe rupture, retinal detachment and full thickness macular hole surgery. Additional exclusion criteria include glaucoma, increased cup to disc ratio, retinal nerve fiber loss on OCT, corneal scar and cataract (≥ grade 2). To avoid interrelationship and potential bias instigated between eyes, only the right eye was used for the analysis.

### Ophthalmological and general examination

Clinical examination in the SDSPS were not limited to OCTA, but also included general health measurements, such as height, weight, calculated Body Mass Index (BMI) and blood pressure. Participants were also interviewed with structured questionnaires, obtaining information about their general health status, concurrent systemic medical conditions, past medical history, as well as smoking and drinking history. A history of smoking was defined as having smoked at least 100 cigarettes during their lifetime. A history of drinking was defined as consuming at least one bottle of alcoholic beverage in the past year, thus including both regular and social drinkers.

Ocular examination included measurement of BCVA using LogMAR charts. The posterior segment (fundus) was assessed with binocular indirect ophthalmoscopy, while the anterior segment was assessed using slit lamp biomicroscopy. Cataracts were graded from 1 to 4 for each category present (nuclear, cortical, posterior subcapsular, and anterior subscapular), using the Lens Opacity Classification System III.

### OCTA examination

**Fig. 1:**
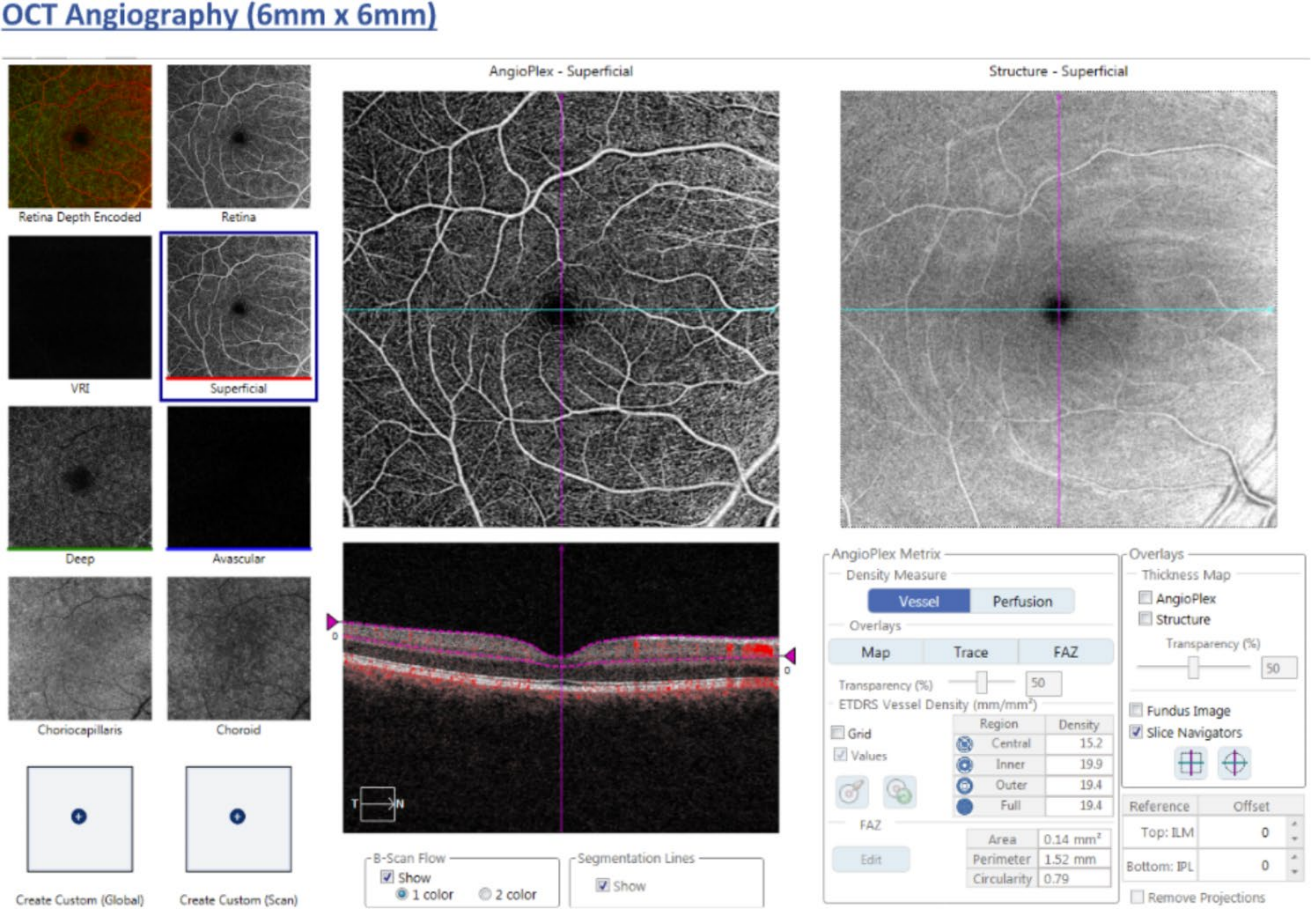
Example OCTA image of the superficial retinal vessels. (Top left image shows an example of the SCP in a 6 × 6 mm OCTA scan from a healthy participant. Top right image shows an OCT fundus scan. The bottom image visualizes the retinal layers, with the top layer identified by arrows and red dotted lines showing the SCP, with red dots in between the dotted lines representing blood flow. The central 1 mm black circle shows the central fovea, with the inner ring (1–3 mm) and outer ring (3–6 mm) surrounding it)

**Fig. 2:**
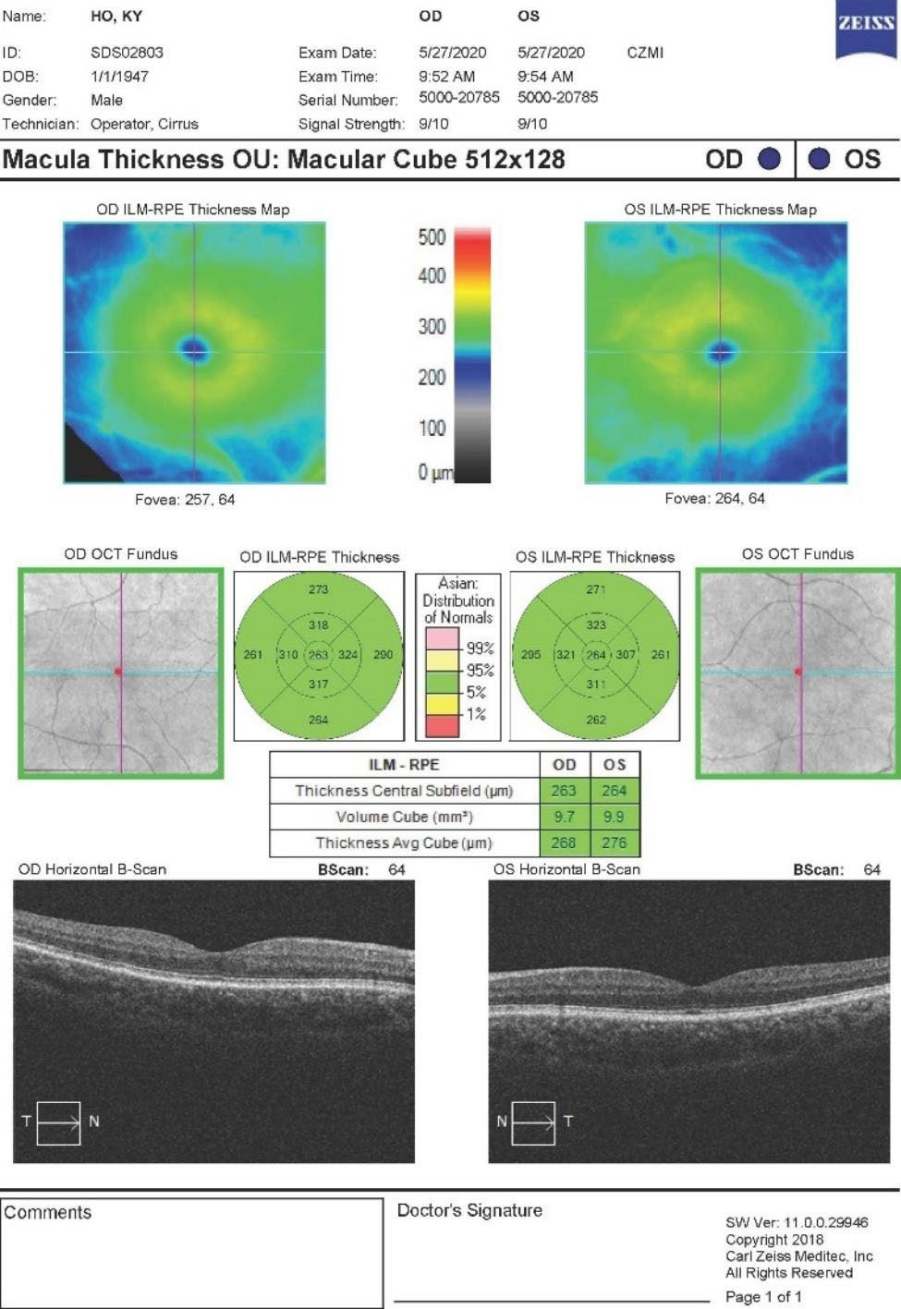
Example image of the macular cube images and ETDRS grid. (Standard output of the Carl Zeiss Cirrus 5000 OCT, 512 × 128 macular cube scan, showing quantitative and qualitative values of the cube details)

OCTA was performed using scan protocol Angioplex 6 × 6 on the Carl Zeiss Cirrus 5000 machine (Carl Zeiss Meditec, Inc, Dublin, CA, USA), covering a 6 × 6 mm square centered on the fovea and using multiple B-scans to distinguish red blood cell movement from static surrounding tissues, through an optical microangiography algorithm with a real-time eye-tracking system [12, 13]. All examinations were performed by trained clinical staff and image quality was assessed by the operator immediately afterwards. Built-in software on the machine was used to quantify MSVD in mm^− 1^, defined as the total length of perfused vasculature (in mm) from 3 μm below the inner limiting membrane (ILM) to 15 μm below the inner plexiform layer (IPL) within the scanned area (in mm^2^). The Early Treatment of Diabetic Retinopathy Study (ETDRS) grid was used to assess vessel densities in the 4 quadrants (nasal, temporal, superior and inferior) in the inner and outer rings as defined by the ETDRS grid. The inner ring was defined as the 1-3 mm ring area, while the outer ring was the 3-6 mm ring area, with the 1 mm area defined as the central foveal area. The cube average thickness (CAT) assessed the average thickness from the ILM to the retinal pigment epithelium (RPE), measured through macular scanning with a 512 × 128 model (128 horizontal B-scans, at 512 A-scans per B-scan). Figures 1 and 2 demonstrate examples of the results obtained from the scan protocol.

### Statistical analysis

Continuous variables were presented as mean ± standard deviation. Categorical variables were presented as frequencies (%). Univariate and multivariate analysis were performed to assess systemic factors and OCTA parameters associated with BCVA. Variables with a p value < 0.10 from univariate analysis were entered into a stepwise multiple linear regression model, with final variables included based on the lowest Akaike information criterion (AIC). Multicollinearity was checked through the variance inflation factor (VIF) scores. P values of < 0.05 were identified as statistically significant. R Studio version 4.1.0 was used for all the statistical analyses.

## Results

From a total of 4774 participants who were all the adults aged 50 and above, 586 were excluded, with 218 having poor quality scans and BCVA LogMAR ≤ 0.8, as this was the standard exclusion criteria proposed in many previous studies involving OCTA and ocular pathologies. Furthermore, further exclusion would be applied for patients with macular or retinal pathologies, thus would still allow the normative values and parameters of OCTA to be assessed. Other subjects excluded include 103 with retinal/macular pathologies and 84 had previous macular or retinal surgeries. Those with previous cataract surgery were included in our analysis. 181 subjects were excluded due to incomplete/poor quality scans, leaving 4188 eyes from 4188 subjects for our analysis. The mean age (in years) of our subjects was 63.51 ± 7.62, with 1450 (34.6%) being male. The mean BMI was 24.31 ± 3.79 kg/m^2^ and the mean systolic and diastolic blood pressure (SBP and DBP) was 135.31 ± 18.94mmHg and 81.91 ± 11.13mmHg respectively. There were 437 (10.4%) subjects with a smoking history, and 329 (7.9%) subjects with a drinking history. (Table 1)

**Table 1:** Baseline demographic and clinical characteristics of the subjects included

	Mean	Standard deviation
Number of eyes	4188	
Age	63.51	7.62
Gender (male)	1450 (34.6%)	
Body mass index (BMI, kg/m^2^)	24.31	3.79
Systolic blood pressure (mmHg)	135.31	18.94
Diastolic blood pressure (mmHg)	81.91	11.13
Smoking history	437 (10.4%)	
Drinking history	329 (7.9%)	
Hypertension	2543 (60.7%)	
Diabetes mellitus	287 (6.9%)	
Hyperlipidemia	790 (18.9%)	
BCVA LogMAR	0.06	0.12
CAT (mm)	270.86	15.74
CST (mm)	249.24	25.82
Central volume (mm^3^)	9.75	0.57
FAZ area (mm^2^)	0.27	0.37
FAZ circularity	0.63	0.12
MSVD Central ring (mm^− 1^)	5.49	3.57
MSVD Inner ring (mm^− 1^)	14.34	4.19
MSVD Outer ring (mm^− 1^)	14.86	3.57
MSVD whole en face image (mm^− 1^)	14.48	3.60
MCPD Central ring (mm^− 1^)	0.16	0.08
MCPD Inner ring (mm^− 1^)	0.39	0.07
MCPD Outer ring (mm^− 1^)	0.42	0.06
MCPD whole en face image (mm^− 1^)	0.41	0.06

BCVA LogMAR = best corrected visual acuity Logarithm of the Minimum Angle of Resolution; CAT = cube average thickness; CST = central subfield thickness; FAZ = foveal avascular zone; MSVD = mean superficial vessel density, MCPD = mean capillary perfusion density

There were 2543 (60.7%) subjects with hypertension (HT), defined as an SBP of ≥ 140 mmHg, DBP of ≥ 90 mmHg, or self-reported HT. There were 287 (6.9%) and 790 (18.9%) of subjects who self-reported a diagnosis of diabetes mellitus (DM) and hyperlipidaemia (HL) respectively. The mean BCVA LogMAR was 0.06 ± 0.12, while the mean FAZ area was 0.27 ± 0.37mm^2^, while the mean FAZ circularity was 0.63 ± 0.12. The MSVD of the included population was 14.48 ± 3.60 mm^− 1^, and 5.49 ± 3.57 mm^− 1^, 14.34 ± 4.19 mm^− 1^, 14.86 ± 3.57 mm^− 1^ in the central, inner and outer rings respectively. (Table 1)

Univariate and multivariate analysis demonstrated age (standardized regression coefficient (β) = 0.32, p < 0.001), being male (β = -0.089, p < 0.001), BMI (β = 0.039, p = 0.006), FAZ area (β = 0.039, p = 0.01) and circularity β=-0.034, p = 0.02), and MSVD in the outer ring (β =-0.51, p < 0.001) being associated with BCVA, with increasing age, BMI and FAZ area showing associations with a lower VA and being male, and having a higher MSVD, FAZ circularity in the outer ring being associated with increased VA. (Table 2)

**Table 2:** Univariate and multivariate on OCTA and systemic associations with BCVA

	Univariate linear regression		Multivariate linear regression	
	Standardized Regression coefficient	P value	Standardized Regression coefficient	P value
Age	0.413	< 0.001***	0.321	< 0.001***
Gender (male)	-0.047	0.003**	-0.089	< 0.001***
BMI	0.058	< 0.001***	0.039	0.006*
Systolic blood pressure	0.117	< 0.001***	0.015	0.428
Diastolic blood pressure	-0.006	0.709		
Smoking history	-0.018	0.240		
Drinking history	-0.019	0.219		
Hypertension	0.103	< 0.001***	0.003	0.895
Diabetes mellitus	0.045	0.003**	-0.003	0.825
Hyperlipidemia	0.060	< 0.001***	0.015	0.295
CAT	-0.132	< 0.001***	-0.124	0.599
CST	0.004	0.80		
CV	-0.131	< 0.001***	0.126	0.593
FAZ area	0.093	< 0.001***	0.039	0.010*
FAZ circularity	-0.137	< 0.001***	-0.034	0.024*
mVD central area	-0.211	< 0.001***	0.032	0.862
mVD inner ring	-0.292	< 0.001***	-0.181	0.348
mVD outer ring	-0.362	< 0.001***	-0.513	< 0.001***
mCPD central area	-0.208	< 0.001***	-0.064	0.728
mCPD inner ring	-0.284	< 0.001***	-0.295	0.128
mCPD outer ring	-0.353	< 0.001***	-0.186	0.219

BMI = body mass index; CAT = cube average thickness; CST = central subfield thickness; FAZ = foveal avascular zone; mVD = mean vessel density; mCPD = mean capillary perfusion density

*=p values < 0.05, **=p values < 0.01, ***=p values < 0.001

Subgroup analysis of the individual quadrants in Table 3 revealed VD in the outer nasal and temporal quadrant being negatively associated with BCVA LogMAR with statistical significance (β = -0.226 and − 0.259, p < 0.001 respectively), while capillary perfusion density (CPD) in the outer superior quadrant was positively associated with BCVA LogMAR with statistical significance (β = -0.123, p = 0.016). This reveals that as the VD in the outer nasal and temporal quadrant, and MCPD in the outer superior quadrant increases, BCVA increases.

**Table 3:** Subgroup analysis showing the associations between ETDRS and specific VD and CPD regions

	Standardized beta coefficient β [95% CI]	P value
VD in ETDRS regions		
VD central area	0.052	0.778
VD inner nasal quadrant	-0.028	0.669
VD_inner superior quadrant	-0.017	0.773
VD_inner temporal quadrant	-0.045	0.494
VD_inner inferior quadrant	-0.044	0.477
VD_outer nasal quadrant	-0.226	< 0.001***
VD_outer superior quadrant	-0.076	0.122
VD_outer temporal quadrant	-0.259	< 0.001***
VD_outer inferior quadrant	-0.110	0.037*
CPD in ETDRS regions		
CPD_central area	-0.089	0.630
CPD_inner nasal quadrant	-0.083	0.198
CPD_inner superior quadrant	-0.096	0.113
CPD_inner temporal quadrant	-0.073	0.260
CPD_inner inferior quadrant	-0.074	0.238
CPD_outer nasal quadrant	-0.010	0.861
CPD_outer superior quadrant	-0.123	0.016*
CPD_outer temporal quadrant	-0.059	0.247
CPD_outer inferior quadrant	-0.083	0.104

VD = vessel density; ETDRS = early treatment diabetic retinopathy study; CPD = capillary perfusion density

*=p values < 0.05, **=p values < 0.01, ***=p values < 0.001

## Discussion

Although previous OCTA studies have been performed assessing demographic, systemic and ocular alterations on retinal vessel densities in healthy eyes [2, 11], the sample sizes were small, eliciting possible selection bias in the recruitment of participants. This is the first prospective cross-sectional and community-based study of this size providing a normative database on common OCTA parameters in Hong Kong citizens aged 50 and above, while also revealing its associations with BCVA and biomarkers predicting BCVA. Our results show increasing age, being female, having a high BMI, high FAZ area and low FAZ circularity, and low MSVD in the outer ring, specifically the nasal and outer quadrants, and low CPD in the outer superior quadrant being statistically significant biomarkers in predicting a risk of lower VA in this population.

Previous studies have been performed on the associations between age and VA, with a study by Radner et al. revealing the significant threshold for this association to be after the age of 55, with those aged 55–64 having a mean BCVA LogMAR of -0.12 ± 0.06 compared to -0.10 ± 0.05 in those aged 65–74 [14], with 97.5% of eyes from healthy participants aged between 25 and 54 achieving a BCVA LogMAR of -0.1 or lower, compared to 71.25% and 51.25% in those aged between 55 and 64 and 65–74 respectively [14]. This may be due to the increased degenerative processes occurring in the ageing population, with the most prominent one being the development of cataracts. We have therefore excluded significant cataracts from analysis.

Associations between gender and VA have also been reported, such as in the study by Emerole et al., revealing females to have poorer vision than males in a cohort of 2606 participants [15], with the majority of them aged between 40 and 64. A proposed theory for this could be because increased age and subsequent menopause in women results in lower oestrogen levels, with a study by Toker et al. reporting a significantly lower flow velocity (p = 0.02) and higher resistive index (RI) (p = 0.001) in central retinal arteries of post-menopausal women when compared with pre-menopausal women [16], which would affect their visual acuity, due to the higher chances of developing retinal ischemia. RI also decreased, while flow velocity increased, with increasing oestradiol levels, and decreased when testosterone levels were raised in both groups [16]. Indeed, oestrogen has been associated with reduction in cardiovascular risks in premenopausal women, offering protective effects against such as preventing myocardial dysfunction and atherosclerosis. This corresponds to our results, with females actually having a lower central VD and CPD, perhaps as the females in our study were over the age of 50, and thus were more likely in menopause, with lower oestrogen levels.

Associations between a high BMI and visual impairment have also been reported in previous studies performed in Hong Kong, revealing statistically significant associations between the two, with obesity (BMI > = 25) having an adjusted odds ratio (aOR) of 4.00 [95% confidence interval [CI] 1.49–11.41] and hyperlipidemia having an aOR of 3.60 [95% CI 1.13–10.97] in increasing the risks of visual impairment [17].

Few studies assessed the associations between VA and OCTA parameters in healthy eyes, as most of these studies focused on pathological eyes with DR or diabetic macular oedema (DMO). A study by Abdelshafy et al. revealed significant negative correlations between VA and VD in the SCP and DCP, especially in the whole enface region for the superficial region and parafovea region in the DCP [18]. This differs from our results, where only MSVD in outer ring or the parafovea region was associated with VA, instead of the whole enface region. The most likely reason for this could be due to the fact that only healthy eyes without retinal pathologies were assessed our study, and even though this encompassed eyes with a BCVA < = 0.8 logMAR (20/125 Snellen equivalents) they could still be considered healthy, as visual acuity worsens naturally in the aging process. The study by Abdelshafy et al. also assessed eyes with and without DR, although other reasons for the differing findings could be due to the ethnicity of the population resulting in biological variation, along with different inclusion and exclusion criteria, OCTA machine with different algorithms used, and variations in sample size, as only 60 eyes of the Egyptian population were evaluated [18]. Correlations between the superficial FAZ area and VA have also been reported, revealing a negative relationship between the two (r=-0.54, p = 0.03) [19], which is consistent with our findings. However, this study also assessed eyes with retinal pathologies, in particular, central retinal vein occlusion.

Associations between MSVD, MCPD and VA have also been reported in another study by Leng et al., reporting MSVD decreasing with myopia and longer axial length (p = 0.021 and 0.027) [8], which is consistent with our findings. Similarly, another study by Milani et al. revealed myopic eyes being negatively correlated with MSVD in Chinese participants (p < 0.001), with the high myopia group (defined as having a spherical equivalent (SE) > 6 diopters) having an MSVD of 19.64% compared to 25.64% in the control group with no or mild myopia (SE >-3 diopters and < 3 diopters) [20]. The exact mechanism of how myopia and reduced BCVA decreases MSVD and CPD remains unconfirmed, but the general hypothesis is that as the axial length is increased, retinal vessels are similarly straightened and narrowed, reducing the overall blood flow and perfusion [21]. Another proposed mechanism by Wang et al. is that as the eyeball elongates in progressing myopia, the retinal tissue concurrently stretches and thins out, reducing the oxygen demand, leading to reductions in the MSVD and CPD [22]. Despite its unconfirmed mechanism, multiple studies have reported myopic eyes having significant reductions in MSVD and choriocapillaris circulation [23–25].

Out of all the variables associated with BCVA in our multivariate model, the OCTA biomarkers predicting BCVA includes: FAZ area and circularity (β = 0.039 and − 0.034, p = 0.01 and 0.024 respectively), MSVD in the outer ring (β=-0.513, p < 0.001), specifically in the nasal and temporal quadrants (β = -0.226 and − 0.259, p < 0.001 for both), and low CPD in the outer superior quadrant (β= -0.123, p = 0.016), although systemic factors such as age, gender and BMI should also be taken into consideration for both older aged individuals and ophthalmologists. Reasons for these specific quadrants having a significantly lower MSVD and MCPD remains unknown, and warrants further research. However, these specific regions should be given greater attention when reviewing OCTA results.

Our study is the first one assessing the associations between OCTA parameters in specific ETDRS quadrants with BCVA in healthy eyes, as well as in this specific population aged 50 and above. However, our study presents with some limitations. First, selection bias may be present, as subjects participating in this programme are voluntary and may be more risk adversed individuals, while age may also be a confounder for comorbidities and poor visual acuity. Moreover, axial length was not measured in our study, which is an important indicator for visual acuity. OCTA also requires high levels of cooperatively from participants, such as to remain still during the procedure, to reduce motion artifacts and poor quality scans, which may be difficult for older subjects with motor disorders. The DCP was also not assessed in our study, so associations of BCVA with parameters in the DCP could not be examined.

However, the major strength of our study is its large sample size, increasing the power of the study, and increasing the accuracy of our linear regression models. The population-based and prospective nature of the study, and how it specifically focuses on the older population also aids in providing a normative database and ocular and systemic factors influencing the MSVD in this population. Our strict inclusion and exclusion criteria also enabled only healthy eyes to be assessed. We also reported VD in mm^− 1^, instead of measuring the area occupied by perfused vessels divided by the total area (%), because this method has been suggested to improve the accuracy of measurements when quantifying finer vessels [26], by measuring the vessel length per surface area in mm/mm^2^ (mm^− 1^). OCTA scans at the level of the SCP also include large arteries and veins, instead of just assessing retinal vessels and retinal microvasculature, and assessing the total area of perfused vasculature may overestimate retinal tissue perfusion [26], so choosing this method to assess the MSVD is more precise. Therefore, our study adds significant and accurate information to the current knowledge surrounding the normal retinal microvasculature.

In conclusion, our present study revealed the average MSVD in adults aged 50 and above. Increased age, being female, having a high BMI, high FAZ area, low FAZ circularity, low MSVD in the outer ring, specifically the nasal and outer quadrants, and low CPD in the outer superior quadrant are independently significant risk factors for reductions in VA and should be considered in clinical practice and in citizens of this population, while these OCTA parameters can be used as biomarkers to predict deteriorations in VA.

## Data Availability

The datasets used and/or analysed during the current study are available from the corresponding author on reasonable request.

## List of abbreviations

FA: Fluoresceine angiography
OCTA: Optical coherence tomography angiography
SDSPS: Southern District Signature Project Scheme
BCVA: Best corrected visual acuity
BCVA LogMAR: Best corrected visual acuity Logarithm of the Minimum Angle of Resolution
CAT: Cube average thickness
CST: Central subfield thickness
FAZ: Foveal avascular zone
MSVD: Mean superficial vessel density
MCPD: Mean capillary perfusion density
BMI: Body mass index
mVD: Mean vessel density
mCPD: Mean capillary perfusion density
VD: Vessel density
ETDRS: Early treatment diabetic retinopathy study
CPD: Capillary perfusion density
SCP: Superficial capillary plexus
DCP: Deep capillary plexus
RI: Resistive index
